# BIGDATA: A Protocol to Create and Extend a 25-Year Clinical Trial and Observational Data Asset to Address Key Knowledge Gaps in Otitis Media and Hearing Loss in Australian Aboriginal and Non-Aboriginal Children

**DOI:** 10.3389/fped.2022.804373

**Published:** 2022-04-14

**Authors:** Jemima Beissbarth, Heidi C. Smith-Vaughan, Allen C. Cheng, Peter S. Morris, Amanda J. Leach

**Affiliations:** ^1^Menzies School of Health Research, Charles Darwin University, Darwin, NT, Australia; ^2^School of Public Health and Preventive Medicine, Monash University, Melbourne, VIC, Australia; ^3^Infection Prevention and Healthcare Epidemiology Unit, Alfred Health, Melbourne, VIC, Australia; ^4^Royal Darwin Hospital, Darwin, NT, Australia

**Keywords:** otitis media, pneumococcal conjugate vaccines, *Streptococcus pneumoniae*, non-typeable Haemophilus influenzae, nasopharyngeal carriage, middle ear microbiology, antimicrobial resistance, indigenous

## Abstract

**Introduction:**

Otitis media (OM) is a common childhood illness, often resolving without intervention and acute and long-term complications are rare. However, Australian Aboriginal and Torres Strait Islander infants and children experience a high burden of OM and are at high risk of complications (tympanic membrane perforation and chronic infections). Bacterial OM is commonly associated with *Streptococcus pneumoniae*, non-typeable *Haemophilus influenzae*, and *Moraxella catarrhalis*. BIGDATA is a data asset combining over 25 years of microbiology and OM surveillance research from the Ear Health Research Program at Menzies School of Health Research (Northern Territory, Australia), including 11 randomized controlled trials, four cohort studies, eight surveys in over 30 remote communities (including data from Western Australia), and five surveys of urban childcare centers including Aboriginal and Torres Strait Islander and non-Indigenous children. Outcome measures include clinical examinations (focusing on OM), antibiotic prescriptions, pneumococcal vaccination, modifiable risk factors such as smoking and household crowding, and nasopharyngeal and ear discharge microbiology including antimicrobial resistance testing.

**Methods and Analysis:**

The initial series of projects are planned to address the following key knowledge gaps: (i) otitis media prevalence and severity over pre pneumococcal conjugate vaccines (PCVs) and three eras of increasing PCV valency; (ii) impact of increasing valency PCVs on nasopharyngeal carriage dynamics of pneumococcal serotypes, and antimicrobial resistance; (iii) impact of increasing valency PCVs on nasopharyngeal carriage dynamics and antimicrobial resistance of other otopathogens; and (iv) serotype specific differences between children with acute OM and OM with effusion or without OM. These data will be utilized to identify research gaps, providing evidence-based prioritization for ongoing research.

**Ethics and Dissemination:**

Data asset creation and priority analyses were approved by the Human Research Ethics Committee of Northern Territory Department of Health and Menzies School of Health Research (EC00153, 18-3281), the Child and Adolescent Health Service Human Research Ethics Committee and Western Australian Aboriginal Health Ethics Committee. Dissemination will be through peer review publication and conference presentations.

## Highlights

- This expandable data asset created from over 25 years of research includes standardized assessments of the middle ear (video otoscopy and tympanometry), risk factors (household crowding, smoking, breastfeeding, living with young children), microbiological data from nasopharyngeal swabs and ear discharge (pneumococcal serotyping, antimicrobial resistance), and medical record review, and antibiotic use is an invaluable data resource.
- The planned analyses will comprehensively describe the OM trends and otopathogen carriage and resistance prevalence over the period 1992 to 2018, and describe factors increasing or decreasing OM and carriage risk, primarily the impact of three sequential pneumococcal conjugate vaccines on pneumococcal serotype replacement.
- This dataset includes research and health service data only where individual consent was provided, and does not describe other broader trends that may impact OM and carriage such as health policy change (medical management guidelines, workforce engagement, and community wide interventions like mass drug administrations). However, data-linkage and historical records can be used to link trends in OM and otopathogens carriage with policy change, workforce trends, housing stock, and public health interventions.
- Original eligibility criteria for participants varied across the studies according to the condition and outcomes of interest, therefore only surveillance and baseline screening data represent community prevalence of OM.
- Beyond the described priority analyses this resource will be used to improve our understanding of ear disease pathology and best practice treatment (from trials and administrative data) to reduce otitis media prevalence and severity in high-risk populations. This includes data linkage to assess educational outcomes, surgical intervention outcomes, and hearing aid fittings. As we are the only group providing pneumococcal serotype epidemiology data in Australia, we plan to develop a parsimonious surveillance modeling system, and will apply to funding to test this system and extend monitoring of pneumococcal serotype replacement and antimicrobial resistance as new extended valency pneumococcal conjugate vaccines are introduced to the Australian market.

## Introduction

Otitis media (OM) is a common childhood illness globally, with an estimated burden of more than 360 million episodes each year (1). OM is one of the primary reasons for presentations at healthcare facilities and for antibiotic prescriptions in children (2). There are varying disease pathologies, from episodic middle ear effusions to chronic drainage of purulent discharge through a perforation of the tympanic membrane. For most infants and children OM will resolve without treatment or intervention. However, some children are at high risk of more severe OM and long-term negative outcomes. Groups at higher risk for OM include children attending out of home childcare and Indigenous populations worldwide. Recognized risk factors for OM include age (with highest incidence between 6 and 11 months), exposure to tobacco and environmental smoke, reduced breast-feeding, and crowded living conditions (2).

Australia's Northern Territory (NT) faces some unique challenges. There is a vast geography with many widely dispersed small populations, with some residents living in very isolated areas. Multiple languages exist, and there is extensive population mobility linked to cultural and social traditions. Approximately 30% of the NT population are Aboriginal, compared to 3% Australia wide. Aboriginal people in the NT are generally younger, have lower educational attainment, have higher rates of chronic health problems, and live in more crowded housing than non-Aboriginal NT residents. 77% live in remote areas, contributing to difficulties in accessing health care and other services (3). The prevalence and severity of otitis media in these remote communities in northern and central Australia is extraordinarily high. Around 90% of children have some form of OM throughout their early years (including 15% with perforated tympanic membrane) (4–6).

All forms of OM are associated with hearing loss (7). The extreme prevalence of OM among Aboriginal children the NT makes this a significant public health problem. OM associated conductive hearing loss can impact the development of language skills and school preparedness when it occurs in children <4 years of age. However, there are limited audiology data for Aboriginal children in this age group (8, 9). The relationship between OM disease status, hearing loss, and language development in this age group are still being established.

The burden of ear disease in NT Aboriginal children starts in the first weeks of life, driven by early, dense colonization of the nasopharynx with common bacterial respiratory pathogens including *Streptococcus pneumoniae*, non-typeable *Haemophilus influenzae* and *Moraxella catarrhalis* (10). *S. pneumoniae* causes considerable morbidity and mortality worldwide due to its ability to cause a range of mucosal and invasive diseases (including otitis media, sinusitis, conjunctivitis, pneumonia, meningitis and bacteremia) (11). The current pneumococcal conjugate vaccines (PCV) confer protection against up to 13 common serotypes (of the 100 known serotypes) causing invasive pneumococcal disease (IPD). The decrease in nasopharyngeal (NP) carriage of vaccine types (VT) among vaccinees has reduced pneumococcal transmission and rates of IPD in non-vaccinated groups as a result of herd effects (12). Near elimination of NP carriage by vaccine serotypes has also left a niche for non-vaccine types (non-VTs). Most populations have reported NP carriage replacement with non-VTs, as was observed in remote dwelling Australian Aboriginal children (13). Whilst non-VT serotypes are less likely to cause IPD than VTs, they continue to cause mucosal pneumococcal infections (including OM and bronchitis). Higher valency PCV trials are currently in progress (including15PCV, PCV20, PHiD-CV11, PHiD-CV12) (14–16). Carriage serotype epidemiology is needed for selection of vaccines as benefits will be population specific and driven by local serotype distribution and dynamics.

### Objectives

The objective of the BIGDATA project is to use an expandable dataset created from otitis media and pneumococcal surveillance studies spanning decades of research to examine questions that could not previously be addressed using single studies. A series of planned analyses will be used to describe OM and NP carriage and identify research gaps to provide evidence-based prioritization for ongoing research. Looking forward, BIGDATA will be linked with health and education administrative data to understand mid-to long-term outcomes for children with and without OM in early childhood, and impacts of past health policies and programs on OM management and long-term outcomes.

## Methods and Analyses

### The Combined Dataset

The Ear Health Research Program (EHRP) at Menzies School of Health Research has been conducting research relating to OM and respiratory pathogen surveillance in children at high risk of ear disease, specifically Australian Aboriginal and/or Torres Strait Islander children living in remote settings, and children attending childcare in urban settings (both at-risk populations) for almost 30 years. A series of cross-sectional studies, longitudinal studies, and randomized controlled trials (RCTs) have been conducted since the early 1990s. This program of research represents a significant data asset relating to ear health states, risk factors, and bacterial pathogen carriage in the nasopharynx (and middle ears of children with tympanic membrane perforation). Currently, BIGDATA includes 3 birth cohort studies, 11 RCTs, 3 cohort studies, 8 surveillance surveys of remote community children (including children in from all over the NT and Kimberley, Goldfields, Pilbara, Gascoyne, and metropolitan area of Perth in Western Australia) and 5 surveillance surveys of urban children attending child care centers (CCC), spanning 1995 to 2018.

### Data Sources, Management, and Amalgamation

A core group of investigators has maintained the EHRP research, ensuring funding, standardized methods for ear assessments and microbiology, long-term consistency of data collection, including cleaning and storage.

Ears were assessed using (video) pneumatic otoscopy or (video) otoscopy and tympanometry. Minimal change to diagnostic categories were made over the life of the program. Management of diagnostic categories of OM were made according to relevant guidelines at the time of each the study. Specialist referrals (audiology and ENT) were also made according to appropriate guidelines. In addition to otitis media diagnoses, detailed ear observations were recorded including tympanic membrane color, translucency, mobility, position (retraction, neutral or degree of bulging), perforation size and position.

Medical records of consented participants were also reviewed to collect additional data. This included non-study related clinician-diagnosed otitis media, all antibiotic prescriptions likely to influence otitis media and pneumococcal immunization data. Health data not related to OM or antibiotic use were not extracted.

Research nurses conducted standardized interview questionnaires with parents or caregivers regarding recognized risk factors. These included breastfeeding, smoking, family history of otitis media, and household occupancy. Observable general health indicators (runny nose, cough, visible skin problems) and weight and height were also collected as part of some studies. Original study data were collected on hard copy standardized forms for entry into secure electronic data storage systems.

Microbiological analysis of NP and ear discharge (ED) swabs for respiratory pathogens of interest used WHO-recommended standardized specimen collection, transport, storage, culture, identification and serotyping methods (Quellung) (17, 18) and are described in detail in Supplementary Material. Antibiotic sensitivity of bacterial isolates included disc diffusion predominantly by Calibrated Dichotomous Susceptibility (CDS) (19) and some E-test MIC determination.

Almost all studies collected a core set of clinical assessments and microbiological specimens and used the same standardized methods and data storage systems.

Some studies included adults, some audiology testing, some virology. All available clinical, microbiological, and risk factor data for children has been included in the combined dataset. Analyses of combined data for uncommon subgroups and outcomes will be undertaken where feasible to do so. Appropriate inclusion and exclusion criteria will be applied in each analysis.

All original data were subject to quality assurance by a process of searching for missing data, logic checks, and audits against primary data sources. Data from the original studies were assessed for variables common in definition. If a comparable variable existed in multiple studies it was included in the combined dataset. Coded data were checked using the original study data dictionary to ensure coding was consistent and standardized as necessary. Variables that were not collected in multiple studies or had inconsistent definitions were not included. We aim to continue building the data asset by adding data from pre-1995 studies, and current studies after completion (bolstering data from 2015 onwards).

Over time, some children represented in this dataset have participated in more than one trial or survey (multiple enrolments). Special attention was given to the demographic data, particularly HRN (hospital registration number) to identify any child enrolled in multiple studies and across regions in the NT. This process involved looking for exact matches, (given and family name, date of birth), and highly probable matches (allowing for minor errors in spelling and dates). The HRN is a unique identifier used across the NT and is assigned to a child at birth. This was done computationally initially and finalized by manual review. A generated unique identifier enables the HRN and other identifying fields to be removed to prevent reidentification.

The data from these studies were combined to create a dataset of all study visit dates for each individual child and associated data for that visit date. Summary data of the number of individual children, enrolments, ear examinations, nasopharyngeal swabs and ear discharge swabs are presented in Table 1. The age distribution is predominantly younger children, with a median age of 2.5 years at enrolment (range 0–17.9).

**Table 1:** Summary of BIGDATA participants, enrolments, ear examinations and nasopharyngeal swab data.

	**Remote Aboriginal Communities (*N*, %)**	**Urban Childcare Centers (*N*, %)**
Individual children	4,692 (66.6)	2,358 (33.4)
Enrolments	8,323 (75.2)	2,751 (24.8)
Ear examinations (children)	10,989 (65.8)	5,720 (34.2)
Nasopharyngeal swabs	11,992 (63.8)	6,814 (36.2)

Original study designs, study enrolments, age profiles and NP microbiology and ear examination data inclusion are summarized in Table 2, additional information is included in Supplementary Material. Where data are published, publications are referenced in Table 2.

**Table 2:** Summary of original studies.

**Years** **data coll**	**Description**	**Study design (no. visits)** **(No. RAC/** **CCC)**	**no. children (% Indigenous) Age—mean/median [range]**	**Procedures and micro**	
1996–2001	Double-blinded randomized controlled trial of long-term amoxicillin vs. placebo for otitis media with effusion (20–27)	L (1–18) RCT (3)	125 (100%) 4.1/2.8 [0.2–19]	Ears NP ED	Spn Hi Mc Sa
2000	The clinical course of acute otitis media in high-risk Australian Aboriginal children: a longitudinal study (28)	L (1–16) Cohort (1)	31 (100%) 1.6 /1.1 [0.2–5.8]	Ears NP ED	Spn Hi Mc
2000–2001	Randomized Controlled Trial of Amoxycillin for Persistent Nasal Discharge in Rural and Remote Aboriginal children (29, 30)	L (1–7) RCT (1)	34 (100%) 59.6/61.4 [26.2–109]	Ears NP ED	Spn Hi
2001	Topical ciprofloxacin vs. topical framycetin-gramicidin-dexamethasone in Australian aboriginal children with recently treated chronic suppurative otitis media: a randomized controlled trial (31)	L (1–10) RCT (3)	97 (100%) 7.8/7.4 [1.1–15.2] years	Ears NP ED	Spn Hi Mc Sa Ps
2001	Otitis media in young Aboriginal children from remote communities in Northern and Central Australia: a cross-sectional survey (4)	X Surv (29)	709 (100%) 1.5/1.5 [0.05–3.4]	Ears	–
2001–2004	Longitudinal nasopharyngeal carriage study of Indigenous children receiving 7-valent pneumococcal conjugate vaccine (21, 23–27, 32–34)	L (1–22) Cohort (3)	97 (100%) 1.6/1.1 [0.2–8]	Ears NP ED	Spn Hi
2001	Childcare hygiene intervention study in 20 Darwin childcare centers (22, 25, 29, 30, 35, 36)	L (1–15) RCT (20)	456 (11%) 29/29 (7–54)	Ears NP ED	Spn Hi
2002–2004	Cross sectional surveillance of bacterial nasal carriage and antibiotic resistance in Indigenous children & adults from Tiwi communities (33, 39)	X Cohort (4)	545 (100%) 25 [0.8–80] **years**	NP	Spn
2003	Randomized controlled trial of surfactant vs ciprofloxacin or sofradex for resolving discharge in Indigenous children in a Tiwi community	L (1–4) RCT (1)	32 (100%) 8.3/7.9 [4.4–13.2] **years**	Ears NP ED	Spn Hi Pa
2003 PCV7	Cross sectional surveillance of OM and bacterial nasal carriage and antibiotic resistance in Indigenous children from 27 remote communities (13, 25, 33)	X Surv (27)	644 (100%) 18/18 [6-30]	NP ED	Spn Hi Mc
2003–2005	Double-blinded randomized controlled trial of azithromycin vs. amoxicillin for treatment of acute otitis media in 16 communities (25, 26, 35, 36, 40–43)	L (1-3) RCT (16)	320 (100%) 17.5/13 [6-72]	Ears NP ED	Spn Hi Mc
2003	Cross-sectional surveillance of bacterial nasal carriage and antibiotic resistance in children attending 18 urban childcare centers in Darwin and Alice Springs	X Surv (18)	303 (unknown%) 33.6/34 [2.6-66]	NP	Spn
2004	Cross-sectional surveillance of bacterial nasal carriage and antibiotic resistance in children attending 23 urban childcare centers in Darwin and Alice Springs (25)	X Surv (26)	473 (unknown%) 33.7/33.4 [3.7-83.8]	NP	Spn Hi Mc
2005	Cross sectional surveillance of OM and bacterial nasal carriage and antibiotic resistance in Indigenous children from 18 remote communities (13, 33)	X Surv (18)	819 (100%) 34/34 [0-72]	NP	Spn
2005	Cross-sectional surveillance of bacterial nasal carriage and antibiotic resistance in children attending 26 urban childcare centers in Darwin and Alice Springs	X Surv (23)	394 (11%) 35.5/36.4 [3.2-65.1]	NP	Spn
2006- 2011	PneuMum: A randomized controlled trial of pneumococcal polysaccharide vaccination for Aboriginal and Torres Strait Islander mothers to protect their babies from ear disease (44–49)	L (1-4) RCT (4)	225 (100%) birth	Ears NP ED	Spn Hi Mc Sa
2008-2013	A double blind placebo-controlled randomized clinical trial of the use of oral azithromycin in Aboriginal children between 6 months and 30 months of age presenting with asymptomatic acute otitis media without perforation	L (1-3) RCT (14)	149 (100%) 16.2/16.1 [5–30.7]	Ears NP ED	Spn Hi Mc Sa
2008–2011	High Nasopharyngeal Carriage of Non-Vaccine Serotypes in Western Australian Aboriginal People Following 10 Years of Pneumococcal Conjugate Vaccination (50)	X Surv (40)	1500 (100%) 0–101 **years**	NP ED	Spn Hi Mc Sa
2008	Cross sectional surveillance of OM and bacterial nasal carriage and antibiotic resistance in Indigenous children from 2 remote communities (5, 38, 51)	X Surv (2)	54 (100%) 19.6/20.8 [4.3–32.2]	Ears NP ED	Spn Hi Mc Sa
2009–2010	A randomized control trial of the effect of swimming in pools in Aboriginal children between 5 and 12 years of age presenting with tympanic membrane perforation (52)	L (1–2) RCT (2)	92 (100%) 8.8/8.7 [4.9–13.6] **years**	Ears NP ED	Spn Hi Sa Pa
2009	Cross sectional surveillance of OM and bacterial nasal carriage and antibiotic resistance in Indigenous children from 10 remote communities (5, 38, 51)	X Surv (10)	174 (100%) 17.1/17.3 [0.9–40.5]	Ears NP ED	Spn Hi Mc Sa
2010	Cross sectional surveillance of OM and bacterial nasal carriage and antibiotic resistance in Indigenous children from 21 remote communities (5, 6, 38, 51, 53)	X Surv (21)	253 (100%) 24.5/21.1 [2.7–70.2]	Ears NP ED	Spn Hi Mc Sa
2010	Cross-sectional surveillance of OM, bacterial nasal carriage and antibiotic resistance in children attending 24 urban childcare centers in Darwin & Alice Springs;	X Surv (24)	462 (100%) 33.9/35 [0.3–73.1]	Ears NP ED	Spn Hi Sa
2011	Mobile phones for advanced case management of chronic suppurative otitis media (CSOM) in remote Indigenous communities: a pilot randomized controlled trial (54)	L (1–2) RCT (1)	53 (100%) 7.5/7.9 [0.9–15.1] **years**	Ears ED	Spn Hi S a Pa
2011	Cross sectional surveillance of OM and bacterial nasal carriage and antibiotic resistance in Indigenous children from 19 remote communities (5, 6, 38, 51, 53, 55)	X Surv (19)	568 (100%) 37.6/33.9 [0.4–96.3]	Ears NP ED	Spn Hi Mc Sa
2012	Cross sectional surveillance of OM and bacterial nasal carriage and antibiotic resistance in Indigenous children from 12 remote communities (5, 6, 38, 51, 53, 55, 56)	X Surv (12)	276 (100%) 25.6/23.4 [0.7–76.2]	Ears NP ED	Spn Hi Mc Sa
2012	Cross-sectional surveillance of OM, bacterial nasal carriage and antibiotic resistance in children attending 33 urban childcare centers in Darwin and Alice Springs (53)	X Surv (33)	607 (100%) 34.1/34.8 [3.4–60.8]	Ears ED	Spn Hi Sa Ps
2013	Cross sectional surveillance of OM and bacterial nasal carriage and antibiotic resistance in Indigenous children from 14 remote communities (6, 51, 55)	X Surv (14)	371 (100%) 35.9/36.3 [0.16–82.9]	Ears NP ED	Spn Hi Mc Sa
2011–2018	A randomized controlled trial of pneumococcal conjugate vaccines Synflorix and Prevenar13 in sequence or alone in high-risk Indigenous infants (PREV-IX_COMBO): immunogenicity, carriage and otitis media outcomes (53, 56–58)	L (1-5) RCT (5)	172 (100%) 31.7/32 [25-40] **days**	Ears NP ED	Spn Hi Mc Sa

*L, longitudinal (number visits); RCT, randomized controlled trial; X, cross-sectional; Surv, surveillance. RAC, Remote Aboriginal Community; CCC, Childcare centers; NP, nasopharyngeal; ED, ear discharge; Spn, S.pneumoniae; Hi, H.influenzae; Mc, M.catarrhalis; Sa, S.aureus; Pa, P.aeruginosa*.

As per the CONSIDER statement (37) the generation of the consolidated data asset and the planned primary analyses were subject to Ethics approval process, including review by the Aboriginal Ethics sub-committee. Use of the data for analyses beyond those specified will be subject to the same process. The leaders of this body of work have extensive experience of research with Aboriginal communities and people. These proposed analyses are part of a broader research program to reduce OM in Aboriginal children. All studies from which data have been drawn were preceded by extensive community consultation and engagement with relevant stakeholders within the communities. These studies have provided employment opportunities for Aboriginal people as researchers, and community-based employment in remote settings. Through recent training and education opportunities, Aboriginal youth have joined us in the laboratory, processing samples collected as part of this body of work. The ongoing engagement over almost 30 years of up to 50 Aboriginal communities in the NT demonstrates the importance of improving ear health outcomes for individuals and communities. The Menzies Child Health Division First Nations Reference Group, whose members are from around the NT, provides guidance, support and feedback to researchers to ensure research activities and dissemination are culturally appropriate. All research dissemination through peer-review publication and community-based feedback (oral and written) to stakeholders includes appropriate management to ensure individuals and communities are not identifiable unless written informed consent is provided.

### Data Limitations

Whilst this resource represents a comprehensive collection, data for all described fields were not included in every study due to the variation in study designs. Original eligibility criteria for participants varied across the RCTs according to the condition (specific OM diagnosis) and outcomes of interest, such as intervention trials for treatment of acute OM or chronic suppurative OM. Data from these studies will be used when they meet inclusion criteria for each research question. Inclusion and exclusion criteria for unpublished data studies can be accessed upon reasonable request. This dataset only includes research and health service data where individual consent was provided. Currently community-level data relating to other factors that may impact OM and NP carriage, including government initiatives such as swimming pools, housing, trends in primary healthcare services and policy, or widespread antibiotic use or mass drug administration for trachoma are not included in BIGDATA. These data could potentially be linked in the future.

### Research Questions and Hypotheses

This expandable dataset was constructed with the goal of describing long term population trends in prevalence of otitis media and nasopharyngeal carriage, to increase statistical power in analyses, and to compare clinical pathways (specialist services) and quality of life outcomes (vulnerability, school attendance and performance) for children with or without early chronic otitis media. We aim to identify research gaps and inform and guide new research in ear and hearing health preventative and treatment options to improve outcomes for high risk populations. Priority analyses, which have appropriate Ethical approvals, are described below.

(i) Otitis media prevalence and severity (by diagnosis) including unilateral and bilateral disease trends, over time, during pre- pneumococcal conjugate vaccines (PCVs) and three increasing valency PCV eras (PCV7, PCV10, and PCV13), by region and other known predictors (risk factors).

Published surveillance to 2013 indicates a reduction in the prevalence of severe OM (CSOM or any TMP), however, prevalence of other forms of OM has increased such that overall OM prevalence remains unchanged and unacceptably high (4, 38).

#### Aims

To describe prevalence of OM and severe OM over time and explore factors associated with these outcomes.

#### Outcomes

Presence of OM and different OM diagnoses (proportions with 95% CIs) will be reported, per time period and region. Markers of severity (size of perforation, volume of discharge, bilateral disease) will be compared over time and vaccine eras. Regression analyses, corrected for repeated measures in the same participants where relevant, will be used to describe OM associations with modifiable risk factors, age, time, prior antibiotic use, gender and geographic region.

Multilevel mixed effects models will be used, utilizing fixed effects for antibiotics, vaccination, age, gender and year; and random effects for region.

(ii) Impact of increasing valency PCVs (PCV7, PCV10 and PCV13) on nasopharyngeal carriage dynamics of pneumococcal serotypes, and associated antibiotic resistance, and by region and other known predictors (risk factors).

PCV impact on nasopharyngeal carriage has been addressed in specific EHRP study publications, as the vaccines have been introduced (Table 1). However, a comprehensive descriptive analysis including all study data from the non-vaccine studies and pre-vaccine era has not been undertaken.

#### Aims

To describe prevalence of nasopharyngeal pneumococcal carriage, serotype-specific carriage, and resistance over time and explore associated risk predictors.

#### Outcomes

Using all available non-interventional data, we will describe pneumococcal carriage, including serotypes and antibiotic resistance over vaccine eras (pre-PCV, PCV7, PCV10 and PCV13), reporting children positive for pneumococcus, vaccine and non-vaccine serotypes (proportions with 95% CIs) by age, region and vaccine era for comparison (chi-squared test). Regression analyses for risk predictors will use the same approach as above.

(iii) Impact of increasing valency PCVs on nasopharyngeal carriage dynamics of non-pneumococcal respiratory pathogens and antibiotic resistance over time, by region, and other known predictors (risk factors).

#### Aims

To determine carriage prevalence, density and resistance of non-typeable *H. influenzae, M. catarrhalis*, and *S. aureus*, over the vaccine eras.

#### Outcomes

Proportions positive for carriage, proportions resistant to common antimicrobials (particularly β-lactams and macrolides), categorical carriage density and co-colonization will be reported and analyzed as per the pneumococcal/OM analyses.

(iv) Serotype specific differences in nasopharyngeal carriage between children with acute OM and diagnoses of OM with effusion or healthy ears and the impact of increasing valency PCVs on serotype dynamics and other known predictors (risk factors).

#### Aims

In a subset of children (completed primary immunization series, aged 6 month to 5 years) from BIGDATA, with both a nasopharyngeal swab and an ear examination of the same day, describe the vaccine and non-vaccine serotype carriage, and impact of increasing valency PCVs on the serotypes associated with a diagnosis of AOM or no AOM.

#### Outcomes

Proportions of children (with 95%CIs) positive for pneumococci, serotype specific carriage, and vaccine and non-vaccine type carriage will be compared (chi-square) in children with and without AOM. Regression analyses will describe associations with modifiable risk factors, age and time as described above.

### Future Directions

Beyond these initial analyses, other projects are being proposed for these data. Building on the pneumococcal carriage analyses, we aim to develop an efficient remote community and childcare center sentinel surveillance model for pneumococcal serotypes to determine efficient surveillance methods to detect emerging pneumococcal serotypes and resistance, and to improve the efficiency of sampling (by age range, geographic region, and sampling frequency). Modeling methodology is yet to be finalized. Accuracy of surveillance models will be tested in cross-sectional prospective studies across the NT.

Where we have comprehensive monitoring of individual children, we intend to examine the relationship between persistent ear disease and hearing loss in infancy and early childhood with subsequent outcomes such as hearing aid use and ENT surgical procedures. We will also describe ongoing indicators of life trajectory by combining our data with government held administrative data through the SA-NT Government data-linkage program. Data will be analyzed using multivariate regression approach with appropriate link function. Multivariate regression will be fitted between ear diagnoses and education and health outcomes, using children with healthy ears as the reference. Linking Health and Education administrative datasets to BIGDATA will facilitate the long term follow up of children with persistent OM diagnoses. This would enable an assessment of the impact of current treatments on perforations, and whether specialist services including hospitalization, ear surgery for grommets and tympanoplasties, and availability of hearing augmentation impact AEDC (Australian Early Development Census) and NAPLAN (National Assessment Program—Literacy and Numeracy) results, school attendance, maltreatment and later engagement with the justice system.

A cascade analysis approach will be used to identify which forms of OM receive more appropriate management (e.g., antibiotic prescribing guidelines, follow-up schedule, correct prescribing, audiology and specialist referrals met) and any management trends over time. We will also describe the region and age that contribute to better outcomes, and the potential impacts of vaccine and antibiotic prescribing guidelines. These detailed data will be used to identify critical timepoints where disease progression might be halted or reversed, and critical age groups for maximizing the benefits of hearing aids or surgery on language development, social development, and education outcomes.

We anticipate that these analyses will further support policies and programs aimed at reducing disadvantage attributed to early childhood ear and hearing problems. The analyses will also identify priority gaps in knowledge and lead to improved design of future studies aimed at eliminating the contribution that ear and hearing problems make to poor quality of life.

## Discussion

There are currently no ongoing NT or Australian surveillance studies of OM and NP carriage. Creating this combined data asset and extending content with new studies and linkage of administrative data increases its value for all Australians. While new research is vital, it is important that the data we already have is used effectively and thoroughly. We now have data spanning the early life of four cohorts of infants which will be re-analyzed to gain a clearer picture of OM in the first months of life. We would like to look at the relationship between NP carriage serotypes and those causing pediatric invasive pneumococcal disease as the conjugate vaccines are introduced. We intend to combine similar treatment arms across RCTs to determine the impact of different classes of antibiotics on resistance and take advantage of the increased statistical power compared to original studies. These data and analyses will also identify the research gaps are that can be the focus of prospective research studies.

As mentioned, we plan for the BIGDATA data asset to be expanded, both with additional EHRP study data, and through data linkage. Government initiatives in the region which impact health service delivery especially as relates to hearing services and ear surgery are of particular interest. Similarly, the changes in the primary healthcare workforce and service delivery (including outreach and tele-otology programs) are likely to be important. Programs such as the mass drug administrations of azithromycin for trachoma eradication (which have the potential to impact otopathogen carriage and antimicrobial resistance), can also be linked to BIGDATA in the future.

## Conclusions

This combined dataset is a valuable research tool for understanding otitis media and nasopharyngeal pathogen carriage in at risk populations in the Northern Territory. This data asset and the described planned analyses will improve our understanding of ear disease pathology and best practice treatment to reduce otitis media prevalence and severity in high-risk populations. Our overall goal for the BIGDATA analyses is to improve hearing and long-term outcomes (such as education and employment) and to address the challenge of increasing antimicrobial resistance in the region.

## Ethics Statement

The studies involving human participants were reviewed and approved by Human Research Ethics Committee of Northern Territory Department of Health and Menzies School of Health Research. Written informed consent to participate in this study was provided by the participants' legal guardian/next of kin.

## Author Contributions

AL conceived the idea for the creation of the combined dataset. AL, PM, and HS-V are senior investigators on nearly all the research projects. JB managed the data amalgamation and was responsible for the data and wrote this manuscript. All authors made intellectual contribution to the manuscript.

## Funding

Funding for this project was provided through the Menzies Small Grants Scheme [Menzies School of Health Research, Darwin (no grant number)] and the National Health and Medical Research Council (NHMRC) Centre for Research Excellence in Ear and Hearing Health of Aboriginal and Torres Strait Islander children (CRE_ICHEAR; GNT1078557). None of the funders had input into the design, or implementation of studies or intellectual ownership of associated publications. JB is supported by a NHMRC scholarship (GNT1150901) and a Centre of Research Excellence in Ear and Hearing Health of Aboriginal and Torres Strait Islander children scholarship (CRE_ICHEAR, GNT1078557). Original studies suppling data were funded as described in Supplementary Material; with the majority funded by the NHMRC. Other funding sources for original studies include Wyeth, GSK, and Pfizer.

## Conflict of Interest

The authors declare that the research was conducted in the absence of any commercial or financial relationships that could be construed as a potential conflict of interest.

## Publisher's Note

All claims expressed in this article are solely those of the authors and do not necessarily represent those of their affiliated organizations, or those of the publisher, the editors and the reviewers. Any product that may be evaluated in this article, or claim that may be made by its manufacturer, is not guaranteed or endorsed by the publisher.

## Abbreviations

EHRP: Ear Health Research Program
OM: otitis media
RCT: randomized controlled trial
CCC: childcare center
NP: nasopharyngeal/nasopharynx
NVT: non-vaccine type
ED: ear discharge.
