# Supplementary material for: BIGDATA: A Protocol to Create and Extend a 25-Year Clinical Trial and Observational Data Asset to Address Key Knowledge Gaps in Otitis Media and Hearing Loss in Australian Aboriginal and Non-Aboriginal Children
Source: Front Pediatr. 2022 Apr 14;10:804373. doi: 10.3389/fped.2022.804373 (PMC9047683; doi:10.3389/fped.2022.804373)
Supplement: Supplementary file 1 [file Data_Sheet_1.PDF]

Original studies: methods and published results

For each study, where published, the title and abstract from the primary publication has been included. If the study has not been published, the background and method are described. Most of the studies were funded by the National Health and Medical Research Council of Australia (NHMRC). Other funding is described by study. The HREC reference number is for the Human Research Ethics Committee of Northern Territory Department of Health and Menzies School of Health Research (EC 00153). Approvals from additional ethics committees are not described. Clinical trial registration is included where relevant.

P=Primary publication, S=secondary outcomes or use of data

COMIT 1 (**C**hronic **O**titis **M**edia **I**ntervention **T**rial 1)

Compared to placebo, long-term antibiotics resolve otitis media with effusion (OME) and prevent acute otitis media with perforation (AOMwiP) in a high-risk population: a randomized controlled trial[1]

Background: For children at high risk of chronic suppurative otitis media (CSOM), strategies to prevent acute otitis media with perforation (AOMwiP) may reduce progression to CSOM.

Methods: In a double-blind study in northern Australia, 103 Aboriginal infants with first detection of OME were randomised to receive either amoxicillin (50 mg/kg/d BD) or placebo for 24 weeks, or until bilateral aerated middle ears were diagnosed at two successive monthly examinations (success). Standardised clinical assessments and international standards for microbiology were used.

Results: Five of 52 infants in the amoxicillin group and none of 51 infants in the placebo group achieved success at the end of therapy (Risk Difference = 9.6% [95% confidence interval 1.6,17.6]). Amoxicillin significantly reduced the proportion of children with i) perforation at the end of therapy (27% to 12% RD = -16% [-31,-1]), ii) recurrent perforation during therapy (18% to 4% RD = -14% [-25,-2]), and iii) reduced the proportion of examinations with a diagnosis of perforation during therapy (20% to 8% adjusted risk ratio 0.36 [0.15,0.83] p = 0.017). During therapy, the proportion of examinations with penicillin non-susceptible (MIC > 0.1 microg/ml) pneumococci was not significantly different between the amoxicillin group (34%) and the placebo group (40%). Beta-lactamase positive non-capsular H. influenzae (NCHi) were uncommon during therapy but more frequent in the amoxicillin group (10%) than placebo (5%).

Conclusion: Aboriginal infants receiving continuous amoxicillin had more normal ears, fewer perforations, and less pneumococcal carriage. There was no statistically significant increase in resistant pneumococci or NCHi in amoxicillin children compared to placebo children who received regular paediatric care and antibiotic treatment for symptomatic illnesses.

Funding: NHMRC 954608 & 980435; **HREC**: 94/25

P[1] S[1-8]

AOM video study

The clinical course of acute otitis media in high-risk Australian Aboriginal children: a longitudinal study [9]

Background: It is unclear why some children with acute otitis media (AOM) have poor outcomes. Our aim was to describe the clinical course of AOM and the associated bacterial nasopharyngeal colonisation in a high-risk population of Australian Aboriginal children.

Methods: We examined Aboriginal children younger than eight years who had a clinical diagnosis of AOM. Pneumatic otoscopy and video-otoscopy of the tympanic membrane (TM) and tympanometry was done every weekday if possible. We followed children for either two weeks (AOM without perforation), or three weeks (AOM with perforation), or for longer periods if the infection persisted. Nasopharyngeal swabs were taken at study entry and then weekly.

Results: We enrolled 31 children and conducted a total of 219 assessments. Most children had bulging of the TM or recent middle ear discharge at diagnosis. Persistent signs of suppurative OM (without ear pain) were present in most children 7 days (23/30, 77%), and 14 days (20/26, 77%) later. Episodes of AOM did not usually have a sudden onset or short duration. Six of the 14 children with fresh discharge in their ear canal had an intact or functionally intact TM. Perforation size generally remained very small (<2% of the TM). Healing followed by re-perforation was common. Ninety-three nasopharyngeal swabs were taken. Most swabs cultured Streptococcus pneumoniae (82%), Haemophilus influenzae (71%), and Moraxella catarrhalis (95%); 63% of swabs cultured all three pathogens.

Conclusion: In this high-risk population, AOM was generally painless and persistent. These infections were associated with persistent bacterial colonisation of the nasopharynx and any benefits of antibiotics were modest at best. Systematic follow up with careful examination and review of treatment are required and clinical resolution cannot be assumed.

Funding: NHMRC 980435; **HREC**: 00/03

P[9] S(-)

Runny Nose Study (unpublished)

Background: Aboriginal children in the Northern Territory have the highest rates of acute and chronic bacterial respiratory disease ever documented. The incidence of invasive pneumococcal disease, chronic suppurative otitis media, chronic bacterial bronchitis and bronchiectasis is alarming, with little evidence of much improvement over the last 25 years. The association between these diseases and persistent nasal discharge has been noted for many years, but never systematically studied.

Objectives: i) To determine if antibiotics provide any benefit to young Aboriginal children with persistent nasal discharge- an efficacy study. ii) To describe the impact of antibiotics on the rates of transmission of bacterial respiratory pathogens.

Methods: Children with excessive nasal discharge, (defined as presence of discharge or excoriation below the nares, or pooling of discharge in the vestibule of the nose), were eligible to enter the run-in period where placebo is administered for 14 days. Children who reached 60% compliance and still had excessive discharge were eligible to be randomised to receive a) amoxycillin, 50mg/kg/day in 2 divided doses; b) Placebo equivalent to 50mg/kg/day in 2 divided doses. Children were examined fortnightly, with video record of nasal discharge and ear state. Nasopharyngeal swabs for microbiological culture were collected at each examination.

Results: unavailable

Funding: NHMRC 100010; **HREC**: 98/61

P(-) S[10, 11]

CSOM (**C**hronic **S**uppurative **O**titis **M**edia)

Topical ciprofloxacin versus topical framycetin-gramicidin-dexamethasone in Australian aboriginal children with recently treated chronic suppurative otitis media: a randomized controlled trial [12]

Background: Chronic suppurative otitis media (CSOM) affects many children in disadvantaged populations. The most appropriate topical antibiotic treatment in children with persistent disease is unclear.

Methods: Children with CSOM despite standard topical treatment were randomized to 6–8 weeks of topical ciprofloxacin (CIP) versus topical framycetin-gramicidin-dexamethasone (FGD). Otoscopic, audiologic, and microbiologic outcomes were measured using standardized assessments and blinding.

Results: Ninety-seven children were randomized. Ear discharge failed to resolve at the end of therapy in 70% children regardless of allocation [risk difference =-2%; (95% CI: -20 to 16)]. Healing of the tympanic membrane occurred in one of 50 children in the CIP group and none of 47 children in the FGD group. Severity of discharge failed to improve in more than 50% children in each group, and mean hearing threshold (38 dB and 35 dB) and proportion of children with greater than 25 dB hearing loss (98% and 88%) were not significantly different between the CIP and FGD groups. Side effects were rare.

Conclusions: This study showed a similarly low rate of improvement or cure in children with persistent CSOM for both CIP and FGD topical therapies. Complications and side effects were insufficient to cease therapy or inform prescribing of either therapy.

Funding: NHMRC 033924; **HREC**: 99/67

P[12] S(-)

PROMPT (**Pr**evention of **O**titis **M**edia with **P**revenar and **T**raining)

Otitis media in young Aboriginal children from remote communities in Northern and Central Australia: a cross-sectional survey [13]

Background: Middle ear disease (otitis media) is common and frequently severe in Australian Aboriginal children. There have not been any recent large-scale surveys using clear definitions and a standardised middle ear assessment. The aim of the study was to determine the prevalence of middle ear disease (otitis media) in a high-risk population of young Aboriginal children from remote communities in Northern and Central Australia.

Methods: 709 Aboriginal children aged 6–30 months living in 29 communities from 4 health regions participated in the study between May and November 2001. Otitis media (OM) and perforation of the tympanic membrane (TM) were diagnosed by tympanometry, pneumatic otoscopy, and video-otoscopy. We used otoscopic criteria (bulging TM or recent perforation) to diagnose acute otitis media.

Results: 914 children were eligible to participate in the study and 709 were assessed (78%). Otitis media affected nearly all children (91%, 95%CI 88, 94). Overall prevalence estimates adjusted for clustering by community were: 10% (95%CI 8, 12) for unilateral otitis media with effusion (OME); 31% (95%CI 27, 34) for bilateral OME; 26% (95%CI 23, 30) for acute otitis media without perforation (AOMwoP); 7% (95%CI 4, 9) for AOM with perforation (AOMwiP); 2% (95%CI 1, 3) for dry perforation; and 15% (95%CI 11, 19) for chronic suppurative otitis media (CSOM). The perforation prevalence ranged from 0–60% between communities and from 19–33% between regions. Perforations of the tympanic membrane affected 40% of children in their first 18 months of life. These were not always persistent.

Conclusion: Overall, 1 in every 2 children examined had otoscopic signs consistent with suppurative ear disease and 1 in 4 children had a perforated tympanic membrane. Some of the children with intact tympanic membranes had experienced a perforation that healed before the survey. In this high-risk population, high rates of tympanic perforation were associated with high rates of bulging of the tympanic membrane.

Funding: Wyeth; **HREC**: 01/06

P[13] S(-)

PRIORiTI (**Pr**evenar **I**mmunisation for **O**titis media **R**eduction in the **T**iwi **I**slands)

Pneumococcal vaccination and otitis media in Australian Aboriginal infants: comparison of two birth cohorts before and after introduction of vaccination [14]

Background: Aboriginal children in remote Australia have high rates of complicated middle ear disease associated with Streptococcus pneumoniae and other pathogens. We assessed the effectiveness of pneumococcal vaccination for prevention of otitis media in this setting.

Methods: We compared two birth cohorts, one enrolled before (1996–2001), and the second enrolled after introduction of 7-valent pneumococcal conjugate and booster 23-valent polysaccharide vaccine (2001–2004). Source populations were the same for both cohorts. Detailed examinations including tympanometry, video-recorded pneumatic otoscopy and collection of discharge from tympanic membrane perforations, were performed as soon as possible after birth and then at regular intervals until 24 months of life. Analyses (survival, point prevalence and incidence) were adjusted for confounding factors and repeated measures with sensitivity analyses of differential follow-up.

Results: Ninety-seven vaccinees and 51 comparison participants were enrolled. By age 6 months, 96% (81/84) of vaccinees and 100% (41/41) of comparison subjects experienced otitis media with effusion (OME), and by 12 months 89% and 88% experienced acute otitis media (AOM), 34% and 35% experienced tympanic membrane perforation (TMP) and 14% and 23% experienced chronic suppurative otitis media (CSOM). Age at the first episode of OME, AOM, TMP and CSOM was not significantly different between the two groups. Adjusted incidence of AOM (incidence rate ratio: 0.88 [95% confidence interval (CI): 0.69–1.13]) and TMP (incidence rate ratio: 0.63 [0.36–1.11]) was not significantly reduced in vaccinees. Vaccinees experienced less recurrent TMP, 9% (8/95) versus 22% (11/51), (odds ratio: 0.33 [0.11–1.00]).

Conclusion: Results of this study should be interpreted with caution due to potential bias and confounding. It appears that introduction of pneumococcal vaccination among Aboriginal infants was not associated with significant changes in prevalence or age of onset of different OM outcomes or the incidence of AOM or TMP. Vaccinees appeared to experience reduced recurrence of TMP. Ongoing high rates of complicated OM necessitate additional strategies to prevent ear disease in this population.

Funding: Wyeth; **HREC:** 01/10

P[14] S [2, 4-8, 14-16]

CHIPS (**C**hildcare **H**ygiene **I**ntervention **P**roject**S**)

Background: Many children receive out of home care prior to starting school. In developed countries childcare centre attendance has been shown to be the most important modifiable risk factor for respiratory tract infections and Otis media in young children. We aimed to test whether a hygiene training package would reduce transmission and lead to less infections in attendees.

Methods: 20 childcare centers in Darwin and Palmerston were randomised to receive a training package around hygiene and hand washing at the beginning or end of the 6 month intervention package. 456 children aged 0-4 years at the participating centres were enrolled to have fortnightly ear examination, and nasal swabs and hand swabs at the start and end of the study period. A health diary was provided to the parents, and fortnightly phone surveys were conducted to record infections and

antibiotic use. Swabs of toys and changing mats were also taken to assess transmission. Trained assessors monitored hygiene behaviors in centre staff using a standardized method after training.

Results not available

Funding: NHMRC 100009; **HREC**: 99/68

P(-) S[3, 6, 10, 11, 17, 18]

CPPP (Community **P**neumococcal **P**rotection **P**roject)

Epidemiology of nasopharyngeal carriage of respiratory bacterial pathogens in children and adults: cross-sectional surveys in a population with high rates of pneumococcal disease [19]

Background: To determine the prevalence of carriage of respiratory bacterial pathogens, and the risk factors for and serotype distribution of pneumococcal carriage in an Australian Aboriginal population.

Methods: Surveys of nasopharyngeal carriage of Streptococcus pneumoniae, non-typeable Haemophilus influenzae, and Moraxella catarrhalis were conducted among adults (≥16 years) and children (2 to 15 years) in four rural communities in 2002 and 2004. Infant seven-valent pneumococcal conjugate vaccine (7PCV) with booster 23-valent pneumococcal polysaccharide vaccine was introduced in 2001. Standard microbiological methods were used.

Results: At the time of the 2002 survey, 94% of eligible children had received catch-up pneumococcal vaccination. 324 adults (538 examinations) and 218 children (350 examinations) were enrolled. Pneumococcal carriage prevalence was 26% (95% CI, 22-30) among adults and 67% (95% CI, 62-72) among children. Carriage of nontypeable H. influenzae among adults and children was 23% (95% CI, 19-27) and 57% (95% CI, 52-63) respectively and for M. catarrhalis, 17% (95% CI, 14-21) and 74% (95% CI, 69-78) respectively. Adult pneumococcal carriage was associated with increasing age ($p = 0.0005$ test of trend), concurrent carriage of non-typeable H. influenzae (Odds ratio [OR] 6.74; 95% CI, 4.06-11.2) or M. catarrhalis (OR 3.27; 95% CI, 1.97-5.45), male sex (OR 2.21; 95% CI, 1.31-3.73), rhinorrhoea (OR 1.66; 95% CI, 1.05-2.64), and frequent exposure to outside fires (OR 6.89; 95% CI, 1.87-25.4). Among children, pneumococcal carriage was associated with decreasing age ($p < 0.0001$ test of trend), and carriage of non-typeable H. influenzae (OR 9.34; 95% CI, 4.71-18.5) or M. catarrhalis (OR 2.67; 95% CI, 1.34-5.33). Excluding an outbreak of serotype 1 in children, the percentages of serotypes included in 7, 10, and 13PCV were 23%, 23%, and 29% (adults) and 22%, 24%, and 40% (2-15 years). Dominance of serotype 16F, and persistent 19F and 6B carriage three years after initiation of 7PCV is noteworthy.

Conclusions: Population-based carriage of S. pneumoniae, non-typeable H. influenzae, and M. catarrhalis was high in this Australian Aboriginal population. Reducing smoke exposure may reduce pneumococcal carriage. The indirect effects of 10 or 13PCV, above those of 7PCV, among adults in this population may be limited.

Funding: Wyeth; **HREC**: 01/10

P[19] S[15, 19]

CSURE (Ciprofloxacin **vs S**ofradex **U**se in **R**unny **E**ars**)**

Background: Aboriginal children living in remote communities have an unacceptably high burden of chronic suppurative otitis media (CSOM). Hearing loss associated with CSOM is a significant contributing factor to poor education outcomes, language development and employment. Current standard treatment of up to 16 weeks of topical sofradex is arduous, and 70% of children experience treatment failure.

Methods: A randomized assessor-blinded trial of no antibiotic treatment vs framycetin-gramicidin-dexamethasone (Sofradex®) vs ciprofloxacin (Ciloxin®) enrolled 32 children 1:1:1, who had chronic suppurative otitis media, with a perforation size of at least 5% and moderate to profuse discharge. Video otoscopy of ear state, nasopharyngeal and ear swabs were collected at baseline, and days 5, 8 and 15. Discharge from ears was collected to tests for immunologic and bacteriologic markers.

Results unavailable

Funding: Student funded; **HREC**: 03/72

P(-) S(-)

MARSi (Monitoring **A**ntibiotic **R**esistance and **S**erotypes**)**

Emerging pneumococcal carriage serotypes in a high-risk population receiving universal 7-valent pneumococcal conjugate vaccine and 23-valent polysaccharide vaccine since 2001[20]

Background: In Australia in June 2001, a unique pneumococcal vaccine schedule commenced for Indigenous infants; seven-valent pneumococcal conjugate vaccine (7PCV) given at 2, 4, and 6 months of age and 23-valent pneumococcal polysaccharide vaccine (23PPV) at 18 months of age. This study presents carriage serotypes following this schedule.

Methods: We conducted cross sectional surveys of pneumococcal carriage in Aboriginal children 0 to 6 years of age living in remote Aboriginal communities (RACs) in 2003 and 2005. Nasal secretions were collected and processed according to published methods.

Results: 902 children (mean age 25 months) living in 29 communities in 2003 and 818 children (mean age 35 months) in 17 communities in 2005 were enrolled. 87% children in 2003 and 96% in 2005 had received two or more doses of 7PCV. From 2003 to 2005, pneumococcal carriage was reduced from 82% to 76% and reductions were apparent in all age groups; 7PCV-type carriage was reduced from 11% to 8%, and 23PPV-non-7PCV-type carriage from 31% to 25% respectively. Thus non-23PPV-type carriage increased from 57% to 67%. All these changes were statistically significant, as were changes for some specific serotypes. Shifts could not be attributed to vaccination alone. The top 10 of 40 serotypes identified were (in descending order) 16F, 19A, 11A, 6C, 23B, 19F, 6A, 35B, 6B, 10A and 35B. Carriage of penicillin non-susceptible (MIC > = 0.12 μg/mL) strains (15% overall) was detected in serotypes (descending order) 19A, 19F, 6B, 16F, 11A, 9V, 23B, and in 4 additional serotypes. Carriage of azithromycin resistant (MIC > = 2 μg/mL) strains (5% overall), was detected in serotypes (descending order) 23B, 17F, 9N, 6B, 6A, 11A, 23F, and in 10 additional serotypes including 6C.

Conclusion: Pneumococcal carriage remains high (~80%) in this vaccinated population. Uptake of both pneumococcal vaccines increased, and carriage was reduced between 2003 and 2005. Predominant serotypes in combined years were 16F, 19A, 11A, 6C and 23B. Antimicrobial nonsusceptibility was detected in these and 17 additional serotypes. Shifts in serotype-specific carriage suggest a need more research to clarify the association between pneumococcal vaccination and carriage at the serotype level.

Funding: NHMRC 236218; **HREC**: 02/68

P[20] S[6, 15, 20]

AATAAC (Azithromycin vs **A**moxycillin for **T**reatment of **A**OM in **A**boriginal **C**hildren)

Single-dose azithromycin versus seven days of amoxycillin in the treatment of acute otitis media in Aboriginal children (AATAAC): a double blind, randomised controlled trial[21]

Objective: To compare the clinical effectiveness of single-dose azithromycin treatment with 7 days of amoxycillin treatment among Aboriginal children with acute otitis media (AOM) in rural and remote communities in the Northern Territory.

Methods: Aboriginal children aged 6 months to 6 years living in 16 rural and remote communities were screened for AOM. Those diagnosed with AOM were randomly allocated to receive either azithromycin (30mg/kg as a single dose) or amoxycillin (50mg/kg/day in two divided doses for a minimum of 7 days). We used a double-dummy method to ensure blinding. Our study was conducted from 24 March 2003 to 20 July 2005. Primary outcome was failure to cure AOM by the end of therapy; nasal carriage of Streptococcus pneumoniae and non-typeable Haemophilus influenzae (NTHi)

Results: We followed 306 of 320 children (96%) allocated to the treatment groups. Single-dose azithromycin did not reduce (or increase) the risk of clinical failure (50% failure rate [82/165]) compared with amoxycillin (54% failure rate [83/155]) (risk difference [RD], – 4% [95% CI, –15% to 7%]; P=0.504). Compared with amoxycillin, azithromycin significantly reduced the proportion of children with nasal carriage of S. pneumoniae (27% v 63%; RD, –36% [95% CI, –47% to – 26%]; P< 0.001) and NTHi (55% v 85%; RD, – 30% [95% CI, –40% to –21%]; P<0.001). Nasal carriage of S. pneumoniae with intermediate or full resistance to penicillin was lower (but not significantly so) in the azithromycin group (10% v 16%), but this group had significantly increased carriage of azithromycin-resistant S. pneumoniae (10% v 3%; RD, 7% [95% CI, 0.1% to 12%]; P=0.001). Carriage of β-lactamase-producing NTHi was about 5% in both groups.

Conclusion: Although azithromycin reduced nasal carriage of S. pneumoniae and NCHi, clinical failure was high in both treatment groups. The possibility of weekly azithromycin treatment in children with persistent AOM should be evaluated.

Funding: NHMRC 193306; **HREC**: 02/44; **Trial registry:** ACTRN12609000691246

P[21] S[6, 7, 17, 18, 21-24]

PneuMum: a randomised controlled trial of maternal 23-valent pneumococcal polysaccharide vaccination on middle ear disease amongst Indigenous infants

Impact of the 23-valent pneumococcal polysaccharide vaccination in pregnancy against infant acute lower respiratory infections in the Northern Territory of Australia [25]

Background: We assessed maternal 23-valent pneumococcal polysaccharide (23vPPV) vaccine efficacy (VE) against middle ear disease and pneumococcal carriage amongst Australian Indigenous infants.

Methods: In an open label, allocation concealed, outcome-assessor blinded, community stratified, randomised controlled trial, healthy pregnant Indigenous women aged 17–39 years in the Northern Territory of Australia received the 23vPPV (1:1:1) at: 30–36 weeks gestation, birth, or were unvaccinated (ClinicalTrials.gov NCT00714064). Co-primary outcomes were the point prevalences of infant middle ear disease and 23vPPV-type carriage at age 7 months.

Results: The consent rate was 50% (313/632). Among 227 eligible participants randomised, retention rates were 86% (66/77) controls; 89% (67/75) pregnancy vaccinees; 88% (66/75) birth vaccinees. At infant age 7 months, ear disease prevalence was: 71% (47/66) controls, 63% (42/67) pregnancy vaccinees, 76% (50/66) birth vaccinees; and 23vPPV-type carriage was: 26% (17/66) controls, 18% (12/67) pregnancy vaccinees, 18% (12/66) birth vaccinees. For pregnancy vaccinees, VE was 12% (95% CI −12% to 31%) against infant ear disease and 30% (95% CI −34% to 64%) against 23vPPV-type carriage. In a post-hoc analysis, VE against infant ear disease concurrent with carriage of 23vPPV or related types was 51% (95% CI −2% to 76%). There were no serious adverse effects following receipt of the 23vPPV in pregnancy or at birth.

Conclusions: In a high risk population, our study was unable to demonstrate efficacy of 23vPPV in pregnancy against the co-primary outcomes of either all-cause infant ear disease or 23vPPV-type nasopharyngeal carriage at age 7 months. Efficacy against ear disease concurrent with carriage of vaccine related serotypes (a more specific outcome) suggests 23vPPV in pregnancy may complement childhood pneumococcal vaccination programs.

Funding: NHMRC 350499 & 490320; **HREC**: 04/54, 05/52; **Trial registry:** NCT00310349

P[26] S[25-30]

AAAOM (Antibiotics for **A**symptomatic **A**cute **O**titis **M**edia**)**

Background: Around 20% of young Australian Aboriginal children living in remote regions have tympanic membrane perforations. This is the third trial of antibiotic treatment of acute otitis media (AOM) in this population.

Methods: 149 Aboriginal children aged 6 months to 6 years living in 14 rural and remote communities were screened via video otoscopy and those with a diagnosis of asymptomatic AOM were randomised to either two single doses of azithromycin (30mg/kg) a week apart or placebo. The primary outcome was failure to cure AOM at end of therapy (day 14). Nasal swabs were collected to describe carriage of OM pathogens.

Results: unavailable

Funding: NHMRC 436023; **HREC**: 07/19 08/56; **Trial registry:** ACTRN12608000424303

P(-) S(-)

Monitoring carriage of *Streptococcus pneumoniae* among Aboriginal children and adults in Western Australia [31]

Background: Invasive pneumococcal disease (IPD) continues to occur at high rates among Australian Aboriginal people. The seven-valent pneumococcal conjugate vaccine (7vPCV) was given in a 2-4-6-month schedule from 2001, with a 23-valent pneumococcal polysaccharide vaccine (23vPPV) booster at 18 months, and replaced with 13vPCV in July 2011. Since carriage surveillance can supplement IPD surveillance, we have monitored pneumococcal carriage in western Australia (WA) since 2008 to assess the impact of the 10-year 7vPCV program.

Methods: We collected 1,500 nasopharyngeal specimens from Aboriginal people living in varied regions of WA from August 2008 until June 2011. Specimens were cultured on selective media. Pneumococcal isolates were serotyped by the Quellung reaction.

Results: Streptococcus pneumoniae, Haemophilus influenzae and Moraxella catarrhalis were carried by 71.9%, 63.2% and 63.3% respectively of children <5 years of age, and 34.6%, 22.4% and 27.2% of people ≥5 years. Of 43 pneumococcal serotypes identified, the most common were 19A, 16F and 6C in children <5 years, and 15B, 34 and 22F in older people. 7vPCV serotypes accounted for 14.5% of all serotypeable isolates, 13vPCV for 32.4% and 23vPPV for 49.9%, with little variation across all age groups. Serotypes 1 and 12F were rarely identified, despite causing recent IPD outbreaks in WA. Complete penicillin resistance (MIC ≥2μg/ml) was found in 1.6% of serotype 19A (5.2%), 19F (4.9%) and 16F (3.2%) isolates and reduced penicillin susceptibility (MIC ≥0.125μg/ml) in 24.9% of isolates, particularly 19F (92.7%), 19A (41.3%), 16F (29.0%). Multi-resistance to cotrimoxazole, tetracycline and erythromycin was found in 83.0% of 23F isolates. Among non-serotypeable isolates 76.0% had reduced susceptibility and 4.0% showed complete resistance to penicillin.

Conclusions: Ten years after introduction of 7vPCV for Aboriginal Australian children, 7vPCV serotypes account for a small proportion of carried pneumococci. A large proportion of circulating serotypes are not covered by any currently licensed vaccine.

Funding: NHMRC 545232; **HREC**: 08/83

P[31] S(-)

SSSOM (**S**wimming **S**tudy for Chronic **S**uppurative **O**titis **M**edia)

Impact of swimming on chronic suppurative otitis media in Aboriginal children: a randomised controlled trial [32]

Objectives: To measure the impact of 4 weeks of daily swimming on rates of ear discharge among Aboriginal children with a tympanic membrane perforation (TMP) and on the microbiology of the nasopharynx and middle ear.

Methods: A randomised controlled trial involving 89 Aboriginal children (aged 5–12 years) with a TMP, conducted in two remote Northern Territory Aboriginal communities from August to December 2009. Intervention: 4 school weeks of daily swimming lessons (45 minutes) in a chlorinated pool. Main outcome measures: Proportions of children with ear discharge and respiratory and opportunistic bacteria in the nasopharynx and middle ear.

Results: Of 89 children randomly assigned to the swimming or non-swimming groups, 58 (26/41 swimmers and 32/48 non-swimmers) had ear discharge at baseline. After 4 weeks, 24 of 41 swimmers had ear discharge compared with 32 of 48 non-swimmers (risk difference, -8% (95% CI, -28% to 12%). There were no statistically significant changes in the microbiology of the nasopharynx or middle ear in swimmers or non-swimmers. Streptococcus pneumoniae and nontypeable Haemophilus influenzae were the dominant organisms cultured from the nasopharynx, and H. influenzae, Staphylococcus aureus and Pseudomonas aeruginosa were the dominant organisms in the middle ear. Conclusions: Swimming lessons for Aboriginal children in remote communities should be supported, but it is unlikely that they will substantially reduce rates of chronic suppurative otitis media and associated bacteria in the nasopharynx and middle ear. However, swimming was not associated with increased risk of ear discharge and we found no reason to discourage it.

Funding: NHMRC Student funds; **HREC**: 09/53; **Trial registry:** ACTRN12613000634774

P[32] S(-)

MOPUP (MObile **P**hones **to improve U**ntreated **P**erforations)

Can mobile phone multimedia messages and text messages improve clinic attendance for Aboriginal children with chronic otitis media? A randomised controlled trial [33]

Aim: Does phone multimedia messages (MMS) to families of Indigenous children with tympanic membrane perforation (TMP): (i) increase clinic attendance; (ii) improve ear health; and (iii) provide a culturally appropriate method of health promotion?

Methods: Fifty-three Australian Aboriginal children with a TMP living in remote community households with a mobile phone were randomised into intervention (n = 30) and control (n = 23) groups. MMS health messages in local languages were sent to the intervention group over 6 weeks.

Results: Primary outcome: there was no significant difference in clinic attendance, with 1.3 clinic visits per child in both groups (mean difference −0.1; 95% confidence interval (CI) −1.1, 0.9; P = 0.9). Secondary outcomes: (i) there was no significant change in healed perforation (risk difference 6%; 95% CI −10, 20; P = 0.6), middle ear discharge (risk difference −1%; 95% CI −30, 30; P = 1.0) or

perforation size (mean difference 3%; 95% CI −11, 17; P = 0.7) between the groups; (ii) 84% (95% CI 60, 90) in the control and 70% (95% CI 50, 80) in the intervention group were happy to receive MMS health messages in the future. The difference was not significant (risk difference −14%; 95% CI −37, 8; P = 0.3).

Conclusions: Although there was no improvement in clinic attendance or ear health, this randomised controlled trial of MMS in Indigenous languages demonstrated that MMS is a culturally appropriate form of health promotion. Mobile phones may enhance management of chronic disease in remote and disadvantaged populations**.**

Funding: Australian Government Department of Health and Ageing Hearing Loss Prevention Program; **HREC**: 08/82; **Trial registry:** ACTRN12610000972022

P[33] S(-)

MARSii & iii (Monitoring **A**ntibiotic **R**esistance and **S**erotypes and Ears**)**

Reduced middle ear infection with non-typeable Haemophilus influenzae, but not Streptococcus pneumoniae, after transition to 10-valent pneumococcal non-typeable H. influenzae protein D conjugate vaccine [34]

Background: In October 2009, 7-valent pneumococcal conjugate vaccine (PCV7: PrevenarTM Pfizer) was replaced in the Northern Territory childhood vaccination schedule by 10-valent pneumococcal Haemophilus influenzae protein D conjugate vaccine (PHiD-CV10; Synflorix™ GlaxoSmithKline Vaccines). This analysis aims to determine whether the reduced prevalence of suppurative otitis media measured in the PHiD-CV10 era was associated with changes in nasopharyngeal (NP) carriage and middle ear discharge (ED) microbiology in vaccinated Indigenous children.

Methods: Swabs of the NP and ED were collected in remote Indigenous communities between September 2008 and December 2012. Swabs were cultured using standardised methods for otitis media pathogens. Children less than 3 years of age and having received a primary course of 2 or more doses of one PCV formulation and not more than one dose of another PCV formulation were included in the primary analysis; children with non-mixed single formulation PCV schedules were also compared.

Results: NP swabs were obtained from 421 of 444 (95 %) children in the PCV7 group and 443 of 451 (98 %) children in the PHiD-CV10 group. Non-mixed PCV schedules were received by 333 (79 %) and 315 (71 %) children, respectively. Pneumococcal (Spn) NP carriage was 76 % and 82 %, and non-typeable Haemophilus influenzae (NTHi) carriage was 68 % and 73 %, respectively. ED was obtained from 60 children (85 perforations) in the PCV7 group and from 47 children (59 perforations) in the PHiD-CV10 group. Data from bilateral perforations were combined. Spn was cultured from 25 % and 18 %, respectively, and NTHi was cultured from 61 % and 34 % respectively (p = 0.008).

Conclusions: The observed reduction in the prevalence of suppurative OM in this population was not associated with reduced NP carriage of OM pathogens. The prevalence of NTHi-infected ED was lower in PHiD-CV10 vaccinated children compared to PCV7 vaccinated children. Changes in clinical severity may be explained by the action of PHiD-CV10 on NTHi infection in the middle ear. Randomised controlled trials are needed to answer this question.

P[34] S[34-39]

MARSiv & v (Monitoring **A**ntibiotic **R**esistance and **S**erotypes and Ears**)**

General health, otitis media, nasopharyngeal carriage and middle ear microbiology in Northern Territory Aboriginal children vaccinated during consecutive periods of 10-valent or 13-valent pneumococcal conjugate vaccines [36]

Objectives: This study aims to monitor the prevalence of suppurative otitis media in remote Indigenous communities after introduction of 13-valent pneumococcal conjugate vaccine (PCV13) in October 2011. We previously reported a decline in suppurative OM following replacement of PCV7 by 10-valent pneumococcal Haemophilus influenzae protein D conjugate vaccine (PHiD-CV10) in October 2009.

Methods: We continued regular surveillance in remote Indigenous communities between February 2010 and August 2013. This analysis reports the general health, otitis media (OM), nasopharyngeal (NP) carriage and middle ear microbiology in children less than 36 months of age who received a primary course of at least two doses ofPHiD-CV10 or PCV13, and not more than one dose of another pneumococcal vaccine.

Results: Mean ages of 511 PHiD-CV10- and 140 PCV13-vaccinated children were 19 and 13 months, respectively. Most children received 3-dose non-mixed PCV schedules. At the time of assessment, general health was poor and prevalence of risk factors was high in both groups: overall, around 14% of children had scabies, 20% had impetigo, 59% had runny nose and 39% had cough. Average household size was 8 persons, and 60% of the mothers smoked. Bilaterally normal middle ears were detected in 10% and 7%, respectively. OM with effusion (OME), almost all bilateral, was diagnosed in 52% and 50%, any suppurative OM (acute OM or any tympanic membrane perforation [TMP]) in 37% and 41%, and TMP in 14% and 12%, respectively. Children in the PCV13 group had significantly less NP carriage of combined Streptococcus pneumoniae (Spn) and non-typeable Haemophilus influenzae (NTHi) (62% versus 51%) but significantly more polymicrobial (Spn and NTHi) middle ear cultures (12% versus 43%), and significantly less Staphylococcus aureus-positive middle ears (40% versus 7%). Although NP carriage of pneumococcal serotype 19A was low in the PCV13 group, serotypes 19F and 23F persist.

Conclusions: The general health, particularly ear health, of little children in remote Australian Indigenous communities remains in crisis. In particular, transition to PCV13 did not show substantial further improvement in ear health. Possible vaccine-related differences in microbiology, including potential beneficial effects of PHiD-CV10 on NTHi infection, need to be further evaluated in randomised trials.

P[34, 36] S[34-40]

MARS CCC (Monitoring **A**ntibiotic **R**esistance and **S**erotypes and Ears in **C**hild **C**are **C**entres**)**

Background: Children attending out of home care experience high rates of respiratory infections and otitis media. *Streptococcus pneumoniae* is one of the main pathogens associated with otitis media. In 2000 a pneumococcal conjugate vaccine was licensed in Australia for infant use. In 2001 a program began for Aboriginal infants only to receive this vaccine at 2, 4 and 6 months of age. This was expanded to all Australian infants in 2005.

Method: To monitor the impact of PCVs on the nasal carriage of *S.pneumoniae* and other respiratory pathogens in children attending urban child care centres in Darwin and Alice Spring (NT, Australia) children aged 0-6 were in enrolled in 2003-5, 2010 and 2012. Nasal swabs were collected and stored according to standard methods. Pneumococci identified by morphology and optochin sensitivity were serotyped using the Quellung method and antimicrobial resistance was determined by CDS. Ear examination by video otoscopy was conducted in the 2010/12 surveys only.

Results not available

All MARSii-v Funding: NHMRC 545232, GSK, Pfizer; **HREC**: 08/83

P(-) S[37]

Pneumococcal conjugate vaccines PREVenar13 and SynflorIX in sequence or alone in high-risk Indigenous infants (PREV-IX_COMBO): protocol of a randomised controlled trial [41]

Background: Otitis media (OM) starts within weeks of birth in almost all Indigenous infants living in remote areas of the Northern Territory (NT). OM and associated hearing loss persist from infancy throughout childhood and often into adulthood. Educational and social opportunities are greatly compromised. Pneumococcus and non-typeable Haemophilus influenzae (NTHi) are major OM pathogens that densely colonise the nasopharynx and infect the middle ear from very early in life. Our hypothesis is that compared to current single vaccine schedules, a combination of vaccines starting at 1 month of age, may provide earlier, broadened protection.

Methods and analyses: This randomised outcome assessor, blinded controlled trial will recruit 425 infants between 28 and 38 days of age and randomly allocate them (1:1:1) to one of three pneumococcal conjugate vaccine (PCV) schedules: Synflorix at 2, 4, 6 months of age, Prevenar13 at 2, 4 and 6 months of age, or an investigational schedule of Synflorix at 1, 2 and 4 months plus Prevenar13 at 6 months of age. The blinded primary outcomes at 7 months of age are immunogenicity of specific vaccine antigens (geometric mean concentration (GMC) and proportion of participants with above threshold GMC of 0.35 µg/L). Secondary outcomes at all timepoints are additional immunogenicity measures and proportion of participants with nasopharyngeal carriage of vaccine-type pneumococci and NTHi, and any OM, including any tympanic membrane perforation. Parental interviews will provide data on common risk factors for OM.

Funding: NHMRC; **HREC**: 1395 **Trial registry:** ACTRN12610000544077 and NCT01174849

P[42] S[43, 44]

References

1. Leach, A.J., et al., *Compared to placebo, long-term antibiotics resolve otitis media with effusion (OME) and prevent acute otitis media with perforation (AOMwiP) in a high-risk population: a randomized controlled trial.* BMC Pediatr, 2008. **8**: p. 23.
2. Binks, M.J., et al., *Viral-bacterial co-infection in Australian Indigenous children with acute otitis media.* BMC Infect Dis, 2011. **11**: p. 161.
3. Smith-Vaughan, H.C., et al., *Measuring nasal bacterial load and its association with otitis media.* BMC Ear Nose Throat Disord, 2006. **6**: p. 10.
4. Marsh, R.L., et al., *Molecular characterisation of pneumococcal serotype 16F: Established predominant carriage and otitis media serotype in the 7vPCV era.* Vaccine, 2007. **25**(13): p. 2434-6.
5. Leach, A.J., et al., *Immunogenicity for 16 serotypes of a unique schedule of pneumococcal vaccines in a high-risk population.* Vaccine, 2008. **26**(31): p. 3885-91.
6. Jacups, S.P., P.S. Morris, and A.J. Leach, *Haemophilus influenzae type b carriage in Indigenous children and children attending childcare centers in the Northern Territory, Australia, spanning pre- and post-vaccine eras.* Vaccine, 2011. **29**(16): p. 3083-8.
7. Smith-Vaughan, H.C., et al., *Dominance of Haemophilus influenzae in ear discharge from Indigenous Australian children with acute otitis media with tympanic membrane perforation.* BMC Ear Nose Throat Disord, 2013. **13**(1): p. 12.
8. Smith-Vaughan, H.C., et al., *Bacteria and viruses in the nasopharynx immediately prior to onset of acute lower respiratory infections in Indigenous Australian children.* Eur J Clin Microbiol Infect Dis, 2018. **37**(9): p. 1785-1794.
9. Gibney, K.B., et al., *The clinical course of acute otitis media in high-risk Australian Aboriginal children: a longitudinal study.* BMC pediatrics, 2005. **5**(1): p. 16-16.
10. Stubbs, E., et al., *Streptococcus pneumoniae and noncapsular Haemophilus influenzae nasal carriage and hand contamination in children: a comparison of two populations at risk of otitis media.* Pediatr Infect Dis J, 2005. **24**(5): p. 423-8.
11. Leach, A.J., et al., *Comparison of nasal swabs with nose blowing for community-based pneumococcal surveillance of healthy children.* J Clin Microbiol, 2008. **46**(6): p. 2081-2.

12. Leach, A.J., et al., *Topical ciprofloxin versus topical framycetin-gramicidin-dexamethasone in Australian aboriginal children with recently treated chronic suppurative otitis media: a randomized controlled trial.* Pediatr Infect Dis J, 2008. **27**(8): p. 692-8.
13. Morris, P.S., et al., *Otitis media in young Aboriginal children from remote communities in Northern and Central Australia: a cross-sectional survey.* BMC Pediatr, 2005. **5**: p. 27.
14. Mackenzie, G.A., et al., *Pneumococcal vaccination and otitis media in Australian Aboriginal infants: comparison of two birth cohorts before and after introduction of vaccination.* BMC Pediatr, 2009. **9**: p. 14.
15. Marsh, R., et al., *The nonserotypeable pneumococcus: phenotypic dynamics in the era of anticapsular vaccines.* Journal of clinical microbiology, 2010. **48**(3): p. 831-835.
16. Balloch, A., et al., *Comparison of anti-pneumococcal antibodies in cord blood from Australian indigenous and Gambian neonates and the implications for otitis media.* The Pediatric infectious disease journal, 2014. **33**(4): p. e116-e120.
17. Smith-Vaughan, H.C., et al., *Absence of an important vaccine and diagnostic target in carriage- and disease-related nontypeable Haemophilus influenzae.* Clin Vaccine Immunol, 2014. **21**(2): p. 250-2.
18. Smith-Vaughan, H.C., et al., *Geographic consistency in dominant, non-typeable Haemophilus influenzae genotypes colonising four distinct Australian paediatric groups: a cohort study.* Pneumonia (Nathan), 2016. **8**: p. 13.
19. Mackenzie, G.A., et al., *Epidemiology of nasopharyngeal carriage of respiratory bacterial pathogens in children and adults: cross-sectional surveys in a population with high rates of pneumococcal disease.* BMC Infect Dis, 2010. **10**: p. 304.
20. Leach, A.J., et al., *Emerging pneumococcal carriage serotypes in a high-risk population receiving universal 7-valent pneumococcal conjugate vaccine and 23-valent polysaccharide vaccine since 2001.* BMC Infect Dis, 2009. **9**: p. 121.
21. Morris, P.S., et al., *Single-dose azithromycin versus seven days of amoxycillin in the treatment of acute otitis media in Aboriginal children (AATAAC): a double blind, randomised controlled trial.* Med J Aust, 2010. **192**(1): p. 24-9.
22. Hare, K.M., et al., *Swab transport in Amies gel followed by frozen storage in skim milk tryptone glucose glycerol broth (STGGB) for studies of respiratory bacterial pathogens.* J Microbiol Methods, 2010. **81**(3): p. 253-5.
23. Marsh, R.L., et al., *Quantitative PCR of ear discharge from Indigenous Australian children with acute otitis media with perforation supports a role for Alloiococcus otitidis as a secondary pathogen.* BMC Ear Nose Throat Disord, 2012. **12**: p. 11.
24. Hare, K.M., et al., *Random colony selection versus colony morphology for detection of multiple pneumococcal serotypes in nasopharyngeal swabs.* The Pediatric infectious disease journal, 2008. **27**(2): p. 178-180.
25. Binks, M.J., et al., *Impact of the 23-valent pneumococcal polysaccharide vaccination in pregnancy against infant acute lower respiratory infections in the Northern Territory of Australia.* Pneumonia (Nathan), 2018. **10**: p. 13.
26. Binks, M.J., et al., *PneuMum: Impact from a randomised controlled trial of maternal 23-valent pneumococcal polysaccharide vaccination on middle ear disease amongst Indigenous infants, Northern Territory, Australia.* Vaccine, 2015. **33**(48): p. 6579-87.
27. Andrews, A., *PneuMum: A randomised controlled trial protocol of pneumococcal polysaccharide vaccination for Aboriginal and Torres Strait Islander mothers to protect their babies from ear disease.* 2010.
28. Johnston, V., et al., *Maternal smoking and smoking in the household during pregnancy and postpartum: findings from an Indigenous cohort in the Northern Territory.* Med J Aust, 2011. **194**(10): p. 556-9.
29. Binks, M.J., et al., *Cord blood vitamin D and the risk of acute lower respiratory infection in Indigenous infants in the Northern Territory.* Med J Aust, 2016. **204**(6): p. 238.
30. McHugh, L., et al., *Birth outcomes in Aboriginal mother-infant pairs from the Northern Territory, Australia, who received 23-valent polysaccharide pneumococcal vaccination during pregnancy, 2006-2011: The PneuMum randomised controlled trial.* Aust N Z J Obstet Gynaecol, 2020. **60**(1): p. 82-87.
31. Collins, D.A., et al., *High nasopharyngeal carriage of non-vaccine serotypes in Western Australian aboriginal people following 10 years of pneumococcal conjugate vaccination.* PloS one, 2013. **8**(12): p. e82280-e82280.
32. Stephen, A.T., A.J. Leach, and P.S. Morris, *Impact of swimming on chronic suppurative otitis media in Aboriginal children: a randomised controlled trial.* Med J Aust, 2013. **199**(1): p. 51-5.

33. Phillips, J.H., et al., *Can mobile phone multimedia messages and text messages improve clinic attendance for Aboriginal children with chronic otitis media? A randomised controlled trial.* J Paediatr Child Health, 2014. **50**(5): p. 362-7.
34. Leach, A.J., et al., *Reduced middle ear infection with non-typeable Haemophilus influenzae, but not Streptococcus pneumoniae, after transition to 10-valent pneumococcal non-typeable H. influenzae protein D conjugate vaccine.* BMC Pediatr, 2015. **15**: p. 162.
35. Leach, A.J., et al., *Otitis media in children vaccinated during consecutive 7-valent or 10-valent pneumococcal conjugate vaccination schedules.* BMC Pediatr, 2014. **14**: p. 200.
36. Leach, A.J., et al., *General health, otitis media, nasopharyngeal carriage and middle ear microbiology in Northern Territory Aboriginal children vaccinated during consecutive periods of 10-valent or 13-valent pneumococcal conjugate vaccines.* Int J Pediatr Otorhinolaryngol, 2016. **86**: p. 224-32.
37. Beissbarth, J., et al., *Recommendations for application of Haemophilus influenzae PCR diagnostics to respiratory specimens for children living in northern Australia: a retrospective re-analysis.* BMC Res Notes, 2018. **11**(1): p. 323.
38. Beissbarth, J., et al., *Use of the 10-valent pneumococcal Haemophilus influenzae protein D conjugate vaccine (PHiD-CV10) in an Australian Indigenous paediatric population does not alter the prevalence of nontypeable Haemophilus influenzae without the protein D gene.* Vaccine, 2019. **37**(30): p. 4089-4093.
39. Harris, T.M., et al., *Culture of non-typeable Haemophilus influenzae from the nasopharynx: Not all media are equal.* J Microbiol Methods, 2017. **137**: p. 3-5.
40. Wigger, C., et al., *A comparison of flocked nylon swabs and non-flocked rayon swabs for detection of respiratory bacteria in nasopharyngeal carriage in Australian Indigenous children.* J Microbiol Methods, 2019. **157**: p. 47-49.
41. Leach, A.J., et al., *Pneumococcal conjugate vaccines PREVenar13 and SynflorIX in sequence or alone in high-risk Indigenous infants (PREV-IX_COMBO): protocol of a randomised controlled trial.* BMJ Open, 2015. **5**(1): p. e007247.
42. Leach, A.J., et al., *Interchangeability, immunogenicity and safety of a combined 10-valent pneumococcal Haemophilus influenzae protein D conjugate vaccine (Synflorix) and 13-valent-PCV (Prevenar13) schedule at 1-2-4-6 months: PREVIX_COMBO, a 3-arm randomised controlled trial.* Vaccine X, 2021. **7**: p. 100086.
43. Leach, A.J., et al., *Otitis media outcomes of a combined 10-valent pneumococcal Haemophilus influenzae protein D conjugate vaccine and 13-valent pneumococcal conjugate vaccine schedule at 1-2-4-6 months: PREVIX_COMBO, a 3-arm randomised controlled trial.* BMC Pediatr, 2021. **21**(1): p. 117.
44. Beissbarth, J., et al., *Nasopharyngeal carriage of otitis media pathogens in infants receiving 10-valent non-typeable Haemophilus influenzae protein D conjugate vaccine (PHiD-CV10), 13-valent pneumococcal conjugate vaccine (PCV13) or a mixed primary schedule of both vaccines: A randomised controlled trial.* Vaccine, 2021. **39**(16): p. 2264-2273.

Microbiological methods

Swab collection, storage and transport

Np swabs (cotton- or rayon-tipped, aluminium shafted superseded cotton tipped wooden shaft swabs) are collected by inserting the swab into nose at an angle parallel to the roof of the mouth, at a depth of half the distance from the earlobe to anterior nostril, holding in position for a count of five seconds with gentle rotation if possible. The quality of this collection is recorded; Good (swab inserted into Np for 5 seconds; Fair (swab inserted only partially and briefly into the nose); Poor (skin just below the nose external – discharge visible); Very poor (skin just below the nose external – no discharge visible). If Np sampling was not possible deep nasal swabs were attempted, and as a last resort nasal secretion collected on a tissue via nose-blowing were swabbed. The swabs are placed inside a 1ml cryovial containing 1ml STGGB (skim milk, tryptone, glucose, glycerol broth) (Gibson, 1986; O'Brien, 2001) Swabs are either placed upright directly into the dry LN2 shipper, or kept cold on wet ice for up to 4 hours before placing in the ultra-low temperature (ULT) freezer (-80degC) or dry LN2 shipper. Swabs stored in dry LN2 shippers are transported to the laboratory in the shipper where they are moved directly to ULT storage. Prior to availability of dry shippers swabs may have been stored at -20°C prior to long term ULT storage.

Ear swabs are collected if ear discharge was observed, by inserting a swab (cotton- or rayon-tipped, aluminium shafted) into the canal and as close as possible to the site of the tympanic membrane perforation (if perforation large enough and under direct vision), with this information recorded as a measure of swab quality; Good (swab collected through TM); Fair (swab collected from deep in canal); Poor (collected discharge visible at canal entrance); Very poor (only external discharge collected). Ear swabs are transported and stored in the same manner as Np swabs.

Primary culture

Np swabs in STGGB were thawed, mixed and 10µL aliquots cultured on the following plates (Oxoid, Australia): 5% horse blood agar, chocolate agar, 5% horse blood agar containing colistin and nalidixic acid, and chocolate agar plus bacitracin, vancomycin and clindamycin (BVCCA)(Chapin, 1983). Swabs of ear discharge were plated additionally onto Pseudomonas selective agar and MacConkey agar. Ear discharge swabs which initially cultured swarming Proteus spp. are filtered to enable detection of *H. influenzae*; a 100µL aliquot was placed on a 65µm nitrocellulose filter which had been placed directly onto BVCCA agar. After 10 minutes the filter was removed. Blood plates were incubated at 37°C in 5% CO_2. Pseudomonas plates are incubated at 42°C, and MacConkey plates at 37°C, all in air.

Streptococcus pneumoniae

Presumptive *S. pneumoniae* were identified by colony morphology, dimpled alpha-haemolytic colonies, and optochin sensitivity. The number of initial colonies selected varied by original study, with minimum one colony, plus any morphologically distinct, selected and plated. *S. pneumoniae* are serotyped with immune sera from the Statens Serum Institute of Copenhagen (Denmark). Antimicrobial susceptibilities are determined by the Calibrated Dichotomous Susceptibility (CDS) disc diffusion method. Antibiotics usually tested for *S. pneumoniae* are oxacillin (1µg), penicillin (0.5IU), tetracycline (30µg), erythromycin (5µg), sulphamethoxazole trimethoprim (25µg), chloramphenicol (30µg). Non-susceptibility is defined by an annular radius of < 6mm. Pneumococcal penicillin minimum inhibitory concentrations (MICs) may have been determined by E-test for non-susceptible capsular *S. pneumoniae* isolates according to individual protocols, and MICs for other antibiotics may have been determined.

Non-typeable Haemophilus influenzae (NTHi)

Presumptive NTHi were identified on the basis of colony morphology (greyish, transparent, smooth colonies) on chocolate agar and bacitracin-vancomycin-clindamycin-chocolate agar (BVCCA)(Chapin, 1983). Minimum one arbitrarily selected colony, plus any morphologically distinct were selected for subculture. The isolates are confirmed as NTHi if hemin and nicotinamide adenine dinucleotide (X and V factor) dependent (Oxoid) and coagglutination negative (Haemophilus Phadebact (10557512, Remel). Beta-lactamase production is determined using nitrocephin (Oxoid, Australia). PCR species confirmation to differentiate NTHi from *Haemophilus haemolyticus* (Hh) is not required, we have previously shown that only 0.34% of Np NTHi isolates from this population are misidentified Hh (Beissbarth, 2018).

Moraxella catarrhalis

One presumptive colony, plus any morphologically distinct colonies are selected for subculture. Confirmation of *M. catarrhalis* is determined by production of oxidase and gram-negative diplococci by gram stain. Beta-lactamase production by *M. catarrhalis* will be determined using nitrocephin (Oxoid, Australia).

Staphylococcus aureus

One presumptive colony, plus any morphologically distinct colonies are selected for subculture. *S. aureus* confirmation is by colony morphology and coagulation by latex agglutination (Staphaurex, Remel superseded Staphytect, Fisher Scientific).

Ear examination

Ear diagnoses were determined by paediatricians or trained research nurses. Diagnosis was determined using video otoscopy to view the tympanic membrane and data collected on standardised forms relating to the colour, translucency, and position and mobility of the drum, presence of perforation, and presence of discharge from perforation. Tympanometry was used to aid diagnosis. Diagnostic categories are as follows.

Table 1 Otitis media diagnostic categories

**Acute Otitis Media without perforation (AOMwoP):** The presence of fluid behind the eardrum plus at least one of the following: bulging eardrum, red eardrum, fever, ear pain or irritability. A bulging eardrum and/or ear pain are the most reliable indicators of AOMwoP.
**Acute Otitis Media with Perforation (AOMwiP):** Discharge of pus through a perforation (hole) in the eardrum within the last 6 weeks. The perforation is usually very small (a pinhole) when the eardrum first ruptures. The perforation can heal and re-perforate after the initial onset of AOMwiP.
**Chronic Suppurative Otitis Media (CSOM):** Persistent ear discharge through a persistent perforation (hole) in the eardrum for 6 weeks or more. Importantly, the diagnosis of CSOM is only appropriate if the tympanic membrane perforation is seen and if it is large enough to allow the discharge to flow out of the middle ear space.
**Dry Perforation:** Presence of a perforation (hole) in the eardrum without any signs of discharge or fluid behind the eardrum. Some people also refer to this as inactive CSOM.
**Middle Ear Discharge:** Fluid containing neutrophils (“pus”) originating from the middle ear cavity. Presence of discharge can often be confirmed by pneumatic otoscopy or swabbing.
**Otitis Media with Effusion (OME):** Presence of fluid behind the ear drum without any acute symptoms. OME may be episodic or persistent. A type B tympanogram or reduced mobility of the ear drum are the most reliable indicators of OME.
**Tympanic membrane perforation (TMP):** Any perforation of the tympanic membrane, including AOMwiP, dry perforation and CSOM.
**Otoscopy:** Looking in the ear with a bright light to identify features associated with outer or middle ear disease.

**Pneumatic Otoscopy:** The combination of simple otoscopy with the observation of eardrum movement when air is blown into the ear canal. Pneumatic otoscopy is able to determine mobility of the eardrum. Reduced mobility of an intact eardrum is a good indication of the presence of middle ear fluid.
**Tympanometry:** An electro-acoustic measurement of the stiffness, mass and resistance of the middle ear (more simply described as mobility of the eardrum). This test can be used to describe normal or abnormal middle ear function.

References

Beissbarth, J., Binks, M. J., Marsh, R. L., Chang, A. B., Leach, A. J., & Smith-Vaughan, H. C. (2018). Recommendations for application of Haemophilus influenzae PCR diagnostics to respiratory specimens for children living in northern Australia: a retrospective re-analysis. *BMC Res Notes, 11*(1), 323. doi:10.1186/s13104-018-3429-z

Chapin, K. C., & Doern, G. V. (1983). Selective media for recovery of Haemophilus influenzae from specimens contaminated with upper respiratory tract microbial flora. *J Clin Microbiol, 17*(6), 1163-1165.

Gibson, L. F., & Khoury, J. T. (1986). Storage and survival of bacteria by ultra-freeze. *Letters in Applied Microbiology, 3*(6), 127-129. doi:doi:10.1111/j.1472-765X.1986.tb01565.x

O'Brien, K. L., Bronsdon, M. A., Dagan, R., Yagupsky, P., Janco, J., Elliott, J., . . . Carlone, G. M. (2001). Evaluation of a medium (STGG) for transport and optimal recovery of Streptococcus pneumoniae from nasopharyngeal secretions collected during field studies. *J Clin Microbiol, 39*(3), 1021-1024. doi:10.1128/jcm.39.3.1021-1024.2001
